# Concurrent partnerships and HIV: an inconvenient truth

**DOI:** 10.1186/1758-2652-14-13

**Published:** 2011-03-15

**Authors:** Helen Epstein, Martina Morris

**Affiliations:** 1 Independent consultant, 424 West 144th Street, New York NY 10031, USA; 2 Departments of Sociology and Statistics, Box 354322 University of Washington, Seattle, WA 98195-4322, USA

## Abstract

The strength of the evidence linking concurrency to HIV epidemic severity in southern and eastern Africa led the Joint United Nations Programme on HIV/AIDS and the Southern African Development Community in 2006 to conclude that high rates of concurrent sexual partnerships, combined with low rates of male circumcision and infrequent condom use, are major drivers of the AIDS epidemic in southern Africa. In a recent article in the *Journal of the International AIDS Society*, Larry Sawers and Eileen Stillwaggon attempt to challenge the evidence for the importance of concurrency and call for an end to research on the topic. However, their "systematic review of the evidence" is not an accurate summary of the research on concurrent partnerships and HIV, and it contains factual errors concerning the measurement and mathematical modelling of concurrency.

Practical prevention-oriented research on concurrency is only just beginning. Most interventions to raise awareness about the risks of concurrency are less than two years old; few evaluations and no randomized-controlled trials of these programmes have been conducted. Determining whether these interventions can help people better assess their own risks and take steps to reduce them remains an important task for research. This kind of research is indeed the only way to obtain conclusive evidence on the role of concurrency, the programmes needed for effective prevention, the willingness of people to change behaviour, and the obstacles to change.

## Introduction

In 2006, a Joint United Nations Programme on HIV/AIDS (UNAIDS) and Southern African Development Community (SADC) group of experts concluded that high rates of concurrent - or overlapping - sexual partnerships, combined with low rates of male circumcision and infrequent condom use, are major drivers of the AIDS epidemic in southern Africa [1]. In a recent article in the *Journal of the International AIDS Society*, Larry Sawers and Eileen Stillwaggon attempt to challenge the evidence for the importance of concurrency [2]. Despite the claim that their article represents a "systematic review of the evidence", it is not an accurate summary of the research on concurrent partnerships and HIV, and it contains factual errors concerning the measurement and mathematical modelling of concurrency.

Critical scrutiny of evidence is a welcome and indeed a necessary part of making progress in science, and all empirical studies have limitations and weaknesses that should be acknowledged. However, Sawers and Stillwaggon's article presents a selective reading of the literature, aimed less at clarification than at advancing the authors' own stated belief that all research on concurrency and AIDS in Africa should be stopped. "The continued use of financial and human resources to prove Western preconceptions about African sexuality cannot be justified," they argue. Instead, they recommend that research resources be invested in understanding the role of bed nets, nutrition, other sexually transmitted infections, recreational drug use, homosexuality and "numerous forms of blood exposures." These, Sawers and Stillwaggon claim, are the "drivers of African HIV epidemics ... for which there is substantial epidemiological evidence."

We do not attempt an exhaustive review of Sawers and Stillwaggon's lengthy article here. Many of the points they raise have already been dealt with in previous exchanges on concurrency and HIV in the journal, *AIDS and Behavior*, and interested readers should consult these articles [3-8]. Here, we address the key specific issues they raise that are new, and demonstrate why they are wrong.

## Discussion

### What is concurrency?

The simple definition of concurrency is when someone begins a new sexual partnership before ending a previous sexual partnership. The precise UNAIDS definition is "overlapping sexual partnerships in which sexual intercourse with one partner occurs between two acts of intercourse with another partner" [9]. The definition covers every form of multiple partnerships other than serial monogamy.

Concurrency can be long term, in which the overlaps last for months or years, or short term, in which the overlaps last for hours or days. Long-term concurrencies include cases in which one person has regular sexual intercourse with more than one partner, such as in a formal polygamous marriage involving a man and more than one wife (or a woman with two husbands), and less formal arrangements in which man has two girlfriends, or a wife and a girlfriend, or a woman has two regular boyfriends, etc. The partners may be spatially separated for defined periods, as in the case of a man who has a wife at home and a girlfriend at a gold mine where he works for months at a time. His wife may have a local boyfriend while he is gone, and this would be concurrency, too [10]. Short-term concurrency includes cases in which a man or woman who has regular sexual contact with only one person also has occasional casual, one-off or commercial sex with others.

### Why does concurrency matter?

All types of concurrency share two "network effects" that distinguish them from multiple serially monogamous partners for the purposes of transmission: they remove the protective effect of sequence, as partnerships begun earlier are indirectly exposed to any infections picked up from a later partner; and they reduce the time to secondary transmission because a recently infected person does not need to end one relationship before starting another.

The longer the average duration of overlap, the greater the impact of concurrency on HIV transmission, which is why long-term concurrencies are the focus of most discussion in this field [11]. If a sufficient fraction of the population has long-term ongoing relationships with more than one person, relatively stable connected sexual networks arise, in which each person's risk is determined not only by his (or her) own behaviour, but also by that of all the others in the network. When the duration of concurrency is short, the connectivity of the networks is more transient, and less conducive to rapid spread.

Long term concurrency also creates conditions that take maximum advantage of the high viral load during the "acute phase" in the first few months following infection. Current estimates suggest the per act transmission risk is 10 to 30 times higher during the acute phase than during the long "chronic phase" that follows [12] (see further discussion in the following pages). If someone has concurrent regular partners, and is newly infected by one of them, he (or she) is able to expose the other partner immediately and repeatedly during this acute phase. With serial monogamy, very high rates of partner acquisition would be required to accomplish something similar: a new partner every few weeks, with multiple coital exposures [13]. Because rates of partner acquisition in any general population are not nearly so high [14], most of those who become infected via serial monogamy will have passed through the acute phase by the time they acquire a new partner.

Finally, long-term concurrent relationships, like all long-term partnerships, are often characterized by strong emotional, social and economic ties; numerous studies suggest that condom use in such relationships tends to be much lower [15-18].

### Is concurrency common in populations severely affected by HIV? Yes

Many peer-reviewed studies of representative samples of adults report high rates of concurrency in the severely HIV-affected populations of southern and eastern Africa [10,11,19-23]. Similar findings with representative samples of local or national populations are found in the reports of non-governmental organizations working on HIV prevention [24-26]. Studies also show that within-country variations in HIV prevalence by subgroup are perfectly aligned with the variations in concurrency by subgroup, both in southern Africa and in the US [11,27,28].

There are limitations to these studies, including differences in the measures used, a lack (in all but one case) of published data on the duration of relationship overlap and coital frequency, inconsistent attention to the gender disparity in prevalence, and the inherent problem created by the mismatch between the timing of behaviour measurement (current, or past 12 months) and the timing of HIV infection (potentially much earlier). However, these limitations do not invalidate the finding that, when equivalent and appropriate measures are compared, the prevalence of concurrency is higher in populations with generalized epidemics of HIV, and not just in African countries. However, the limitations do require that extra care be taken when making inferences and comparisons across populations and studies.

Sawers and Stillwaggon do not mention most of the evidence we have cited, and compare studies that use completely different measures of concurrency to support their argument. Their primary evidence that concurrency is not especially common in Africa is presented in their Table One which lists 28 estimates of "concurrency" from different countries and studies. They claim that the table entries are ranked from high to low by estimates for men, but these estimates are not comparable, so cannot be ranked in this way. Some of the estimates are based on cumulative behaviours over the past five years (Adimora 2002, 2007), others over the past one year (Mishra 2009), while still others refer only to concurrencies active on the day of interview (Carael 1995, and Morris and Kretzschmar 2000). Ranking these is analogous to failing to distinguish studies reporting the number of partners in the past day from those reporting the number of partners in the past five years.

Some figures in Table One also appear to be erroneous. For example, the Kapiga and Lugalla (2002) estimate comes from a paper that uses data from the 1996 Tanzania Demographic and Health Survey (DHS), but that DHS did not measure concurrency. Kapiga and Lugalla simply report the number of non-marital "regular" and "casual" non-spousal partners in the past year, and it is not clear how Sawers and Stillwaggon calculate from this the numbers they report in Table One (despite their endnote). It is clear that their estimate does not include polygyny - reported to be 15% of married men aged 15 to 59 years in that DHS [[29]/, Table 5.3]. Just over half of men in this age group are married, so this alone would roughly double the rate of concurrency among men reported by Sawers and Stillwaggon in this table.

In addition, 10 of the estimates in Table One are from the World Health Organization's Global Programme on AIDS studies conducted between 1989 and 1993 (Carael 1995), while 13 are from DHS studies conducted from 2001 to 2006 (Mishra 2009). A decade separates these two sets of studies, during which reductions in risk behaviours have been documented in almost every country listed [30-33]. In short, the estimates in Table One are interesting, but differences in the measurements used and the survey dates render them incomparable. They cannot be used, as Sawers and Stillwaggon do, to create a meaningful rank order.

The one source of data on concurrency that Sawers and Stillwaggon cite uncritically is the DHS, the results of which have only been reported in an unpublished working paper [34]. This suggests they are unfamiliar with the problems that have been identified in the DHS concurrency data. Demographic and health surveys have been conducted in many developing countries since 1984 to obtain representative national data on a wide range of health indicators. The primary focus of these surveys has traditionally been nutrition, fertility and maternal and child health, and they are a unique and valuable resource for international comparisons on these topics. In 1998, the DHS added optional questionnaire modules on knowledge, attitudes and behaviours relevant to HIV/AIDS, and from 2000, it included a module that was intended to collect data on concurrent partnerships in the past 12 months.

Unfortunately the concurrency data have been plagued by a sequence of errors in the questionnaire design. The module used in surveys from 2000 to 2004 failed to collect data on partnership duration for all but the most recent partner. This means that it is only possible to identify concurrency if the most recent partnership started at least 12 months prior to the date of interview, and the data cannot be used to estimate the duration of partnership overlap.

That omission was rectified in 2005, but two other problems remained. One was the way the DHS asked the duration question ("For how long have you had a sexual relationship with this person?"). Since the module failed to ask whether the relationship was still ongoing, the start date could be calculated either from the date of interview, or from the date of last sex. The uncertainty in establishing the start date of a relationship creates uncertainty in whether it overlapped with any others. The other problem was that the module failed to collect data on partnership duration for spouses and cohabiting partners (it is possible to recover the partnership start date from the marital section of the questionnaire, but only if the respondent has had only one spouse or cohabiting partner in his or her lifetime).

These problems appear to have been fixed in 2009, and the DHS from Lesotho that uses the corrected questionnaire module has found very high annual prevalence of concurrency among both men and women [35]. However, the result of the previous errors has been shown to be a downward bias in the estimates of concurrency, with variability both over time (due to the changes in questionnaire design) and across countries (because the sources of bias turn out to vary across countries) [9,36]. This is deeply unfortunate, as it invalidates the DHS estimates of both levels of and trends in concurrency, as well as cross-country comparisons, prior to 2009.

Even without the errors in the questionnaire, however, collecting concurrency data using the DHS is a challenge. The DHS surveys are quite long and repetitive, involving hundreds of questions about a wide range of health and demographic issues. A report of multiple partners in the past year triggers an additional series of about 10 questions about each partner, for up to three partners. The increasing length and complexity of the DHS questionnaire could create an incentive to under-report for both harried interviewers and respondents [37]. In addition, the DHS surveys are conducted in the households of the participants. While efforts are made to establish privacy, a partner, child, relative or neighbour may be in the room or close by.

Both of these factors may exacerbate the tendency to under-report sexual partnerships in the DHS. Shorter surveys, dedicated to the sensitive task of sexual behaviour measurement, have more carefully designed questionnaires, insist on interviewing in private, and are more likely to minimize that bias. This issue is discussed in more detail in the section on qualitative data.

### Does concurrency correlate with HIV risk at the individual level? Yes, when investigators use the right data and methods

Sawers and Stillwaggon list a number of studies that found no correlation between HIV infection and concurrency at the individual level, but all of them contain one or more serious methodological errors [34,38-40]. The most basic error that these studies share is a fundamental logical flaw in the way they attempt to "test" the concurrency hypothesis: using a respondent's concurrency to predict the respondent's own HIV status. Other things being equal, concurrency does not heighten the risk of HIV acquisition for those who practice it: their risk is determined by the number of partners and coital exposures they have, regardless of the order in which they have them. Rather, concurrency heightens risk for the partners of those who practice it: the classic case is the monogamous person whose only risk comes from the fact that his or her partner has another partner. This is why the studies cited by the authors (and some others) find no significant "effect of concurrency" at the individual level: they fail to specify the model correctly. This point has been made in print repeatedly over the past decade [5,41].

Properly designed studies consistently confirm that concurrency is and remains a key driver in populations experiencing generalized epidemics in Africa. The strongest findings come from studies of stable couples that enrol both partners and use biomarkers to measure incident HIV infection, as these can establish whether new infections arise from inside or outside the couple. The fraction of all incident HIV that occurs within stable couples has been estimated from a longitudinal cohort study in Uganda as 71% before ART scaleup, and 57% after [42]. Stable couples can be divided into three categories: concordant negative (NN), discordant (NP or PN), or concordant positive (PP). Incident infection in stable couples therefore comprises two types: in the first, the couple moves from NN to discordant (NP or PN); and in the second, the couple moves from discordant (NP or PN) to PP. Incident infections of the first type, by definition, must come from outside the couple. Incident infections of the second type can come from within or outside the couple.

Six published studies estimate the fraction of incident cases of the first type (NN to NP or PN). Five are longitudinal cohort studies from Uganda and Tanzania that measure incident infection directly, with follow-up periods from one to seven years: these estimate the fraction of new infections in initially concordant negative couples as 42% [43], 50% [44], 63% [45], 78% [46] and 56-75% (depending on the treatment of missing data) [42]. In most of the studies that published sex specific rates, men were much more likely than women to be the incident case [43,44,46,47]. The remaining study uses the BED assay, an antibody test designed to detect recent infection, on a cross-sectional sample of Ugandans, and finds that among married couples, 49% of recently infected individuals had an HIV-negative spouse [48]. In summary, these studies suggest that the fraction of incident cases in stable couples coming from the first type of "outside the couple" infection ranges from 42% to 78%.

Two published studies estimate the fraction of incident cases of the second type (NP or PN to PP), and both use genetic typing to test whether both members of the couple have the same strain of HIV. One, from a very large, longitudinal multi-site trial in Africa, found that among HIV discordant couples in which the negative partner became infected, 29% of the cases could not be linked [47]. Another, from a smaller cross-sectional study of concordant positive couples [49], found that 35% of the cases could not be linked a sample from Lusaka (where HIV prevalence is around 20% [50]), but all of the cases could be linked in a sample from Kigali (where HIV prevalence is around 7% [51]). This latter study is small, but the results are consistent with the prediction that where concurrency is high (Lusaka), incidence attributable to concurrency is also high.

Together, this implies that 60% to 84% of incident infections in stable couples come from outside the partnership. This figure is derived as follows: (fraction of cases of type 1) + (1 - fraction of cases of type 1) * (fraction of cases of type 2). To bound the range, we take the lowest [43] and highest [46] values from the studies with estimates for the type 1 fraction, [43-46,48]and the estimate from the large, longitudinal multi-site trial for the type 2 fraction [47]. These infections must be due to concurrency; the only alternative is non-sexual transmission (an unlikely scenario for the reasons we discuss below).

### Do ethnographic studies of concurrency have any value? Yes

Sawers and Stillwaggon correctly state that ethnographic research does not provide statistically valid estimates of the prevalence of concurrency. However, this is not the purpose of ethnography. In-depth data collection, at the individual, focus group and community level, is most often used to explore meanings, perceptions and attitudes about concurrency in order to support prevention programming, a purpose for which it is uniquely well suited.

For example, ethnographic research has shed light on the different meanings of material exchange within sexual relationships in different contexts. In contrast to formal prostitution, where a given amount of money is exchanged for the performance of a particular sexual act, the "transactional sex" described in numerous studies in southern and eastern Africa often involves the exchange of gifts and money within ongoing, committed relationships. Several authors have described how transactional sex helps explain women's tolerance of a partner's concurrency behaviours and may also motivate women to have other partners themselves [52-55]. Sawers and Stillwaggon dismiss this important body of research, remarking that readers of *The Lancet *would be astonished to read a paper about how women in Western countries also receive candy and flowers from their regular partners. However, Western women seldom cite candy and flowers as primary motivations for engaging in multiple regular partnerships or for tolerating men who do.

In-depth interviews have also been used to investigate the validity of responses on behavioural surveys. The reluctance of respondents to disclose sensitive sexual behaviour information on standard sample surveys is universally recognized by researchers who work in this field, and efforts to assess the magnitude of the downward bias in quantitative surveys through qualitative triangulation has been a mainstay of HIV/AIDS research since the early 1990 s [56].

One particularly large and well-designed study compared the sexual behaviour reports given in survey type interviews to both in-depth interviews and biomarker verification on the same respondents, and concluded: "In-depth interviews seem to be more effective than assisted self-completion questionnaires and face to face questionnaires in promoting honest responses among females with STIs. Participant observation was the most useful method for understanding the nature, complexity, and extent of sexual behaviour" [57].

Qualitative studies of small population samples consistently find that respondents report engaging in concurrent partnerships at rates that are often many times higher than in behavioural surveys [25,58-63]. These findings demonstrate that many respondents are willing to disclose sensitive behaviours in face-to-face interviews, which suggests that it might also be possible to improve disclosure in traditional behavioural survey interviews. This is an active field of research, with findings supporting a range of different approaches, including Audio Computer-Assisted Self Interviewing (ACASI) surveys or ballot box methods to increase privacy [64,65], more interactive interviews to increase rapport between interviewer and respondent [66], and relationship history calendars to improve the accuracy of reporting [67].

The estimates from these small convenience samples cannot be used to infer rates of concurrency in the population, but they can certainly be used to raise questions about the validity of estimates based on survey data. Ignoring this empirical evidence is simply unscientific.

### Does computer modelling support the concurrency hypothesis? Yes

Computer modelling of transmission networks and concurrency is complex and the field has evolved considerably over the past 15 years. The relevant aspects of this history are described briefly in the following paragraphs.

Sawers and Stillwaggon's discussion of concurrency modelling studies ignores all of the progress that has been made in the field since 2000, and makes claims that are categorically untrue. Specifically, their claim that the concurrency effect observed in the early Morris and Kretzschmar models can only be obtained using unrealistic assumptions about such parameters as coital frequency is simply wrong. Three subsequent independent modelling studies, using empirically derived parameters for all inputs, have now shown that concurrency must have played a critical role in the generalized epidemics in Zimbabwe and South Africa [68-70]. Sawers and Stillwaggon cite none of these studies.

Between 1995 and 2000, Morris and Kretzschmar published a series of studies showing that, all other things equal, HIV would spread much more rapidly through a population in which multiple partnerships were concurrent than through one in which all multiple partnerships were serial [71-74]. The purpose of these early papers was to explore and document the mechanisms by which concurrency could influence transmission dynamics since this had not been done with appropriate modelling methods before. These studies did not aim to describe a real-life epidemic. Neither the authors nor those who cite the study as evidence for the importance of concurrency make this claim [3,75]. In order to model a real epidemic, Morris and Kretzschmar would have had to include numerous other variables, including stage-specific transmission rates and vital dynamics (births and deaths). That was not possible with the methods and data available at the time.

Because Morris and Kretzschmar did not include vital dynamics in their model, they were not able to observe the point at which transmission would fall below the reproductive threshold for persistence. That would only be possible if the model had been designed to remove infected cases from the simulated populations; otherwise, the number of infected cases simply increases or remains stable over time. This is why these original simulations could only compare how quickly the infection spread under different scenarios.

It turns out that adding vital dynamics greatly increases the estimated impact of concurrency, because in the "serial monogamy" scenario - but not in the concurrency scenario - most infected individuals die before they can infect at least one other person. This has been shown in subsequent studies to effectively prevent the spread of HIV via serial monogamy [13,68,69]. Thus, the unrealistic parameters that Sawers and Stilwaggon criticize in the early Morris and Kretzschmar studies actually led to an *underestimate*, not an overestimate, of the effect of concurrency in those studies.

Recently, two independent data-driven modelling studies, using realistic estimates for rates of sexual partner acquisition, concurrency, coital frequency and stage-specific infectivity, as well as vital dynamics, have shown that it is not possible to generate an epidemic in Zimbabwe, at levels of partner acquisition reported from 1998 to 2004, without concurrency [68,69].

One of these actually takes the Morris and Kretzschmar model that Sawers and Stillwaggon criticize, and modifies it to incorporate mortality, stage-specific HIV transmission estimates per partnership, and the empirical rates of concurrency observed in a Zimbabwe sexual behaviour survey [68]. The authors found that they were unable to produce an epidemic without having concurrency in the model.

The other study, using newer methods and a similar range of variables, but also accurately representing the observed gender asymmetry in concurrent long- and short-term partnerships in the sexual network, comes to the same conclusion [69]. This study tested four different stage-specific transmission rate estimates taken from the literature [12,76-78] based on one empirical study from Uganda (no such data is available from Zimbabwe, or anywhere else) [78].

A final simulation study came to a similar conclusion using a very different methodology [13]. It employed a deterministic compartmental model to determine what rate of partner change would be needed with serial monogamy and realistic transmission parameters to reproduce the very rapid early rise in prevalence in South Africa. The rate was absurdly high: an average of two new partners per week, with more than seven coital acts per week.

These papers were not yet published when Sawers and Stillwaggon conducted their review of the literature, but the papers' findings fully refute their claim that "In order to generate rapid spread of HIV, mathematical models *require *unrealistic assumptions about frequency of sexual contact, gender symmetry, levels of concurrency, and per-act transmission rates" (emphasis added). Tellingly, the authors did not cite two other sophisticated modelling studies that had already been published and also used more realistic empirical estimates of behaviour. Both studies demonstrated large impacts of concurrency: one finds that it is responsible for about half of the epidemic potential within heterosexual populations in the US, and helps to explain racial disparities in HIV and sexually transmitted infection (STI) prevalence [28]; and the other finds concurrency plays a major role in the epidemic in South Africa, accounting for roughly three-quarters of new infections from 1990-2000 [70].

### Is coital frequency high enough for HIV to propagate via concurrency? Yes

Sawers and Stillwaggon point out that many studies of African populations find "relatively low" rates of coital frequency: perhaps one or two sex acts per week in regular partnerships on average (in fact, this is the average observed in other parts of the world, as well [79,80]). However, during the acute phase, this can still translate into a remarkably high probability of transmission within a given relationship. Analyses of empirical data collected in Uganda [78] suggests that transmission during the acute phase could be as high as 3.6% per sex act, compared with 0.084% per sex act during the long "chronic phase" before AIDS symptoms appear [12].

Using this estimate, if a discordant couple has sex once a week for two months when the infected partner is in the acute phase, the cumulative probability of transmission to the susceptible partner would be 25% (we calculate the likelihood of transmission as equal to [1-(1-β)^c^], where β is the probability of transmission per act and c is the number of sexual acts). This estimate rises to 44% if they have sex twice a week. Note that in the Ugandan study on which the original probabilities per act were calculated, observed coital frequency was 2.5 times per week - which would imply a 53% chance of transmission over two months.

Since the acute phase of infection is so short (estimates range from two to five months in the studies we have cited [12,76-78]), one would need to have a new partner in this time frame for the high acute transmission probability to influence secondary transmission. Except in situations of very high average partner change - higher than any observed in the heterosexual populations in Africa experiencing hyper-epidemics - most of those practicing serial monogamy will risk passing on the virus during the "latent phase" of infection, when viral load and transmission likelihood are much lower. Concurrency, by contrast, enables the virus to take advantage of the acute phase, even when rates of partner change are very low.

### Is polygamy safe? Only if all partners are strictly faithful to the marriage

Formal polygamy is a type of concurrency that ideally should not be risky, as long as no member of the polygamous unit has extramarital partners. Although one ecological study suggested polygamy may not be riskier than monogamy [81], the authors controlled for extra-marital sex in this analysis, in effect removing the concurrency that would be the mechanism by which HIV entered the marriages, polygamous and otherwise. Moreover, numerous individual-level studies have found that being in a polygamous marriage is a risk factor for extra-marital sex and HIV and other STIs [82-89]. Because the risk to one member of a polygamous unit depends upon the behaviour of all the others, faithfulness and/or consistent condom use are especially important for people in polygamous unions.

### Is the concurrency hypothesis based on a "Western preconception about African sexuality"? No

While some Western researchers were already investigating concurrency in the early 1990 s [90,91], the motivation behind Morris's original concurrency models came from Africans. In 1993, she gave a research presentation at Mulago Hospital in Kampala, Uganda. At the time, she was focusing on the epidemiological impact of what is now called "intergenerational sex".

During her talk, a Ugandan man in the audience raised his hand and asked whether her mathematical models included people "having more than one partner at a time". When she said "no," he got up and walked out of the room. After the talk, Morris was taken aside by a Nigerian field supervisor from Uganda's largest AIDS research study who said, "We really think this [meaning overlapping sexual partnerships] is important here." So, this work was motivated not by a "Western preconception" but by a sincere attempt to respond to the expressed concerns of African researchers who wanted to understand why their communities were so severely affected by AIDS.

### How important are non-sexual drivers of the epidemic? Probably not very

Sawers and Stillwaggon argue that research and programme efforts should be concentrated on non-sexual drivers of the epidemic, including the interaction between HIV and malaria and other tropical diseases, intestinal worms, poor nutrition, other sexually transmitted infections, and drug use and other forms of blood exposure. However, a large body of existing research suggests that the share of HIV cases attributable to these causes is small.

The findings from previous research and the epidemiological evidence suggests that the impact of malaria and other tropical diseases on HIV prevalence is, at best, minimal. Even in highly malarious areas, this disease is estimated to account for only 4.8% of cumulative HIV cases since 1990 [92]. Empirically, HIV rates are particularly high in southern African countries where the prevalence of malaria [93], schistosomiasis [94] and malnutrition [95] is low. Data from the most recent WHO report on the Global Burden of Disease [96] show that the sub-Saharan countries with the highest HIV prevalence in the world -- Botswana, Lesotho and Swaziland -- have the lowest rates of mortality due to malaria and tropical diseases in the region. By contrast, in the countries with the highest rates of mortality due to malaria and tropical diseases -- Democratic Republic of the Congo, Congo-Brazzaville and Ghana, where mortality rates from these diseases are 15 times higher than in Botswana, Lesotho and Swaziland -- rates of HIV related mortality are 80% lower. Even at the beginning of the epidemic, it was the wealthiest sectors of sub-Saharan African populations--those least likely to suffer from the untreated effects of these diseases-- that were first infected with HIV[97].

The role of co-factor STIs has also been the focus of considerable previous research, and while many studies show a correlation between STI and HIV prevalence, the evidence of causal impact is much less compelling. A cross-sectional correlation between prevalent STI and HIV may simply reflect the underlying sexual network that spreads both. STIs may heighten the risk of HIV transmission somewhat, but the failure of several randomized trials of STI treatment for HIV prevention suggest to us that STIs are probably not the main driver of HIV infection in Africa [47,98,99].

The role of injections has also been exhaustively studied, and the data do not support the hypothesis of a significant impact on HIV transmission in the region. While injecting drug use is a growing problem in Africa, especially in large coastal cities, it is still uncommon on most of the continent, particularly among the young women who traditionally have been at the highest risk of HIV acquisition [100]. Other forms of parenteral HIV transmission are rare [101], and a systematic, definitive study of this topic concluded that there is no compelling evidence that unsafe injections are a dominant mode of HIV-1 transmission in sub-Saharan Africa [102].

Finally, novel Africa-specific strains of HIV are unlikely to explain the explosive epidemic in the region either, because those strains have appeared in other world regions, where they have in contrast spread very slowly [103-109].

## Conclusions

In order to accelerate HIV prevention in southern Africa, we do need a better understanding of the key epidemic drivers. The hypothesis that concurrency is one of those drivers is consistent with many observed facts, including the findings that: people in the region do not have more partners on average over the course of their lives than people in other world regions do [11]; infection rates are higher in women than in men, a reverse of the pattern seen in the US, Europe and Asia [110]; and many people with few sexual partners, or even only one, are at high risk because they or their partners are linked to a wider sexual network.

Most interventions to raise awareness about the risks of concurrency are less than two years old; few evaluations and no randomized-controlled trials of these programmes have been conducted. Determining whether these interventions can help people better assess their own risks and take steps to reduce them remains an important task for current research, and research is the only way that conclusive evidence on the role of concurrency, the programmes needed for effective prevention, the willingness of people to change behaviour, and the obstacles to change can be obtained.

We don't deny that factors other than concurrency play a role in the sub-Saharan African epidemic; however, the evidence does not support an important role for the drivers that Sawers and Stillwaggon are promoting. Over the three decades since the AIDS pandemic first emerged, the field has been plagued by highly publicized "controversies" driven by ideological advocates, some of whom have proposed that non-sexual drivers associated with poverty explain the extreme disparities in HIV prevalence within and between countries. Poverty and the burden of preventable diseases are profoundly important in their own right and deserve at least the level of attention that the world gives to HIV, but they are not the primary drivers of HIV transmission.

Using the political power of HIV to draw attention to other unethical global health disparities is justified. However, selective presentation of scientific evidence that may slow progress in HIV prevention has no place in that agenda. It is a dangerous distraction, with potentially fatal consequences. Sawers and Stillwaggon's analyses are neither scientifically accurate nor coherent, and their call for an immediate end to all research on concurrency is not a constructive contribution to HIV prevention.

## Competing interests

The authors declare that they have no competing interests.

## Authors' contributions

HE conceived the main arguments of the paper and wrote the first draft. MM made extensive revisions and other intellectual contributions.

## References

[B1] SADC Expert Think Tank Meeting on HIV Prevention in High-Prevalence Countries in Southern Africa 2006 Maseru, Lesotho

[B2] Sawers L Stillwagon E Concurrent sexual partnerships do not explain the HIV epidemics in Africa: a systematic review of the evidence Journal of the International AIDS Society 2010 14 34 10.1186/1758-2652-13-34 20836882 PMC3161340

[B3] Mah TL Halperin DT Concurrent Sexual Partnerships and the HIV Epidemics in Africa: Evidence to Move Forward AIDS and Behavior 2010 14 11 16 10.1007/s10461-008-9433-x 18648926

[B4] Lurie MN Rosenthal S Concurrent Partnerships as a Driver of the HIV Epidemic in Sub-Saharan Africa? The Evidence is Limited AIDS and Behavior 2010 14 17 24 10.1007/s10461-009-9583-5 19488848 PMC3819098

[B5] Morris M Barking up the Wrong Evidence Tree. Comment on Lurie & Rosenthal, "Concurrent Partnerships as a Driver of the HIV Epidemic in Sub-Saharan Africa? The Evidence is Limited" AIDS and Behavior 2010 14 31 33 10.1007/s10461-009-9639-6 19997971 PMC2814034

[B6] Epstein H The mathematics of concurrent partnerships and HIV: a commentary on Lurie and Rosenthal AIDS and Behavior 2010 14 29 30 10.1007/s10461-009-9627-x 19866354

[B7] Mah TL Halperin DT The Evidence for the Role of Concurrent Partnerships in Africa's HIV Epidemics: A Response to Lurie and Rosenthal AIDS and Behavior 2010 14 25 28 10.1007/s10461-009-9617-z 18648926

[B8] Lurie MN Rosenthal S The Concurrency Hypothesis in Sub-Saharan Africa: Convincing Empirical Evidence is Still Lacking. Response to Mah and Halperin, Epstein, and Morris AIDS and Behavior 2010 14 34 37 10.1007/s10461-009-9640-0 20130786 PMC2815263

[B9] UNAIDS Reference Group on Measurement and Modeling HIV: consensus indicators are needed for concurrency Lancet 2010 14 621 622 10.1016/S0140-6736(09)62040-7 19954832

[B10] Lurie MN Williams BG Zuma K Mkaya-Mwamburi D Garnett GP Sweat MD Gittelsohn J Karim SSA Who infects whom? HIV-1 concordance and discordance among migrant and non-migrant couples in South Africa Aids 2003 14 2245 2252 10.1097/00002030-200310170-00013 14523282

[B11] Morris M Epstein H Wawer M Timing is everything: International variations in historical sexual partnership concurrency and HIV prevalence PLoS One 2010 e14092 10.1371/journal.pone.0014092 21124829 PMC2991312

[B12] Pinkerton SD Probability of HIV transmission during acute infection in Rakai, Uganda AIDS and Behavior 2008 14 677 684 10.1007/s10461-007-9329-1 18064559 PMC2614120

[B13] Delva W Pretorius C Vansteelandt S Temmerman M Williams B Serial monogamy and the spread of HIV: how explosive can it get? XVIII International AIDS Conference; Vienna 2010 Abstract WEAC0105

[B14] Wellings K Collumbien M Slaymaker E Singh S Hodges Z Patel D Bajos N Sexual and reproductive health 2 - Sexual behaviour in context: a global perspective Lancet 2006 14 1706 1728 10.1016/S0140-6736(06)69479-8 17098090

[B15] Anderson JE Wilson R Doll L Jones TS Barker P Condom use and HIV risk behaviors among US adults: Data from a national survey Family Planning Perspectives 1999 14 24 28 10.2307/2991553 10029929

[B16] Macaluso M Demand MJ Artz LM Hook EW Partner type and condom use Aids 2000 14 537 546 10.1097/00002030-200003310-00009 10780716

[B17] Manning WD Flanigan CM Giordano PC Longmore MA Relationship Dynamics and Consistency of Condom Use Among Adolescents Perspectives on Sexual and Reproductive Health 2009 14 181 190 10.1363/4118109 19740237 PMC3035359

[B18] Westercamp N Mattson CL Madonia M Moses S Agot K Ndinya-Achola JO Otieno E Ouma N Bailey RC Determinants of Consistent Condom Use Vary by Partner Type among Young Men in Kisumu, Kenya: A Multi-level Data Analysis AIDS and Behavior 2010 14 949 959 10.1007/s10461-008-9458-1 18791819 PMC3673546

[B19] Carter MW Kraft JM Koppenhaver T Galavotti C Roels TH Kilmarx PH Fidzani B "A bull cannot be contained in a single kraal": Concurrent sexual partnerships in Botswana AIDS and Behavior 2007 14 822 830 10.1007/s10461-006-9203-6 17295072

[B20] Colvin M Abdol Karim S Connolly C Hoosen A Ntuli N HIV infection and asymptomatic sexually transmitted infections in a rural South African community. International Journal of STD & AIDS 1998 14 548 550 10.1258/0956462981922683 9764941

[B21] Mbizvo EM Msuya SE Stray-Pedersen B Sundby J Chirenje MZ Hussain A HIV seroprevalence and its associations with the other reproductive tract infections in asymptomatic women in Harare, Zimbabwe Int J STD AIDS 2001 14 524 531 10.1258/0956462011923624 11487393

[B22] Sandoy IF Dzekedzeke K Fylkesnes K Prevalence and Correlates of Concurrent Sexual Partnerships in Zambia AIDS and Behavior 2010 14 59 71 10.1007/s10461-008-9472-3 18841461

[B23] Vissers DCJ Voeten H Urassa M Isingo R Ndege M Kumogola Y Mwaluko G Zaba B de Vlas SJ Habbema JDF Separation of spouses due to travel and living apart raises HIV risk in Tanzanian couples Sexually Transmitted Diseases 2008 14 714 720 10.1097/OLQ.0b013e3181723d93 18520338

[B24] C-Change A Baseline Survey of Multiple and Concurrent Sexual Partnerships Among Basotho Men in Lesotho 2009 Maseru, Lesotho: USAID, AED

[B25] Parker W Connolly C NAMIBIA: A Midterm Household Analysis of Residents from Keetmanshoop, Oshakati, Rundu and Walvis Bay 2007 Windhoek: NawaLife Trust

[B26] Taruberekera N Jafa K Mushayi W Multiple Concurrent Partnerships in Zimbabwe: Determinants and monitoring indicators 2008 Harare, Zimbabwe: Population Services International

[B27] Kenyon C Dlamini S Boulle A White RG Badri M A network-level explanation for the differences in HIV prevalence in South Africa's racial groups African Journal of AIDS Research 2009 14 243 254 10.2989/AJAR.2009.8.3.1.922 25864540

[B28] Morris M Kurth A Hamilton DT Moody J Wakefield S for The Network Modeling Group Concurrent partnerships and HIV prevalence disparities by race: Linking science and public health practice Amer J Pub Health 2009 14 10.2105/AJPH.2008.147835 PMC2679771 19372508

[B29] Bureau of Statistics [Tanzania] Macro International Tanzania Demographic and Health Survey 1996: Final Report 1997 Calverton, Maryland: Bureau of Statistics [Tanzania] and Macro International

[B30] Bloom SS Banda C Songolo G Mulendema S Cunningham AE Boerma JT Looking for change in response to the AIDS epidemic: Trends in AIDS knowledge and sexual behavior in Zambia, 1990 through 1998 Journal of Acquired Immune Deficiency Syndromes 2000 14 77 85 10.1097/00042560-200009010-00011 11064508

[B31] Gouws E Stanecki KA Lyerla R Ghys PD The epidemiology of HIV infection among young people aged 15-24 years in southern Africa Aids 2008 14 S5 S16 10.1097/01.aids.0000341773.86500.9d 19033755

[B32] Gregson S Gonese E Hallett TB Taruberekera N Hargrove JW Lopman B Corbett EL Dorrington R Dube S Dehne K Mugurungi O HIV decline in Zimbabwe due to reductions in risky sex? Evidence from a comprehensive epidemiological review Int J Epidemiol 2010 14 1311 1323 10.1093/ije/dyq055 20406793 PMC2972436

[B33] Central Statistical Office Ministry of Health University of Zambia Measure Evaluation Zambia Sexual Behavior Survey 2009 Lusaka, Zambia: CSO and Measure Evaluation

[B34] Mishra V Bignami-Van Assche S Concurrent Sexual Partnerships and HIV Infection: Evidence from National Population-Based Surveys 2009 Calverton, Maryland: Macro International Inc

[B35] Lesotho Ministry of Health and Social Welfare Lesotho Demographic and Health Survey 2009 2010 Calverton, Maryland, USA: Ministry of Health and Social Welfare and ICF Macro

[B36] Morris M Leslie-Cook A Evaluating concurrent partnership data from the Demographic and Health Surveys (DHS) XVIII International AIDS Conference; Vienna, Austria 2010

[B37] Murray CJL Loakso T Shibuya K Hill K Lopez AD Can we achieve Millennium Development Goal 4? New analysis of country trends and forecasts of under-5 mortality to 2015 Lancet 2007 14 1040 1054 10.1016/S0140-6736(07)61478-0 17889243

[B38] Jewkes R Dunkle K Nduna M Levin J Jama N Khuzwayo N Koss M Puren A Duvvury N Factors associated with HIV sero-positivity in young, rural South African men Int J Epidemiol 2006 14 1455 1460 10.1093/ije/dyl217 17030525

[B39] Kelly R Gray R Valente T Sewankambo N Serwadda D Wabwire-Mangen F Lutalo T Li C Wawer M Concurrent and non-concurrent sexual partnerships and risk of prevalent and incident HIV International AIDS Conference Durban, South Africa 2000

[B40] Lagarde E Auvert B Carael M Laourou M Ferry B Akam E Sukwa T Morison L Maury B Chege J Concurrent sexual partnerships and HIV prevalence in five urban communities of sub-Saharan Africa Aids 2001 14 877 884 10.1097/00002030-200105040-00008 11399960

[B41] Morris M Concurrent Partnerships and Syphilis Persistence: New Thoughts on an Old Puzzle Sex Transm Dis 2001 14 504 507 10.1097/00007435-200109000-00005 11518866

[B42] Gray R Ssempiija V Shelton J Serwadda D Nalugoda F Kagaayi J Kigozi G Wawer MJ The contribution of HIV-discordant relationships to new HIV infections in Rakai, Uganda Aids 2011 in press 10.1097/QAD.0b013e3283448790 PMC4169210 21326076

[B43] Carpenter LM Kamali A Ruberantwari A Malamba SS Whitworth JAG Rates of HIV-1 transmission within marriage in rural Uganda in relation to the HIV sero-status of the partners Aids 1999 14 1083 1089 10.1097/00002030-199906180-00012 10397539

[B44] Serwadda D Gray RH Wawer MJ Stallings RY Sewankambo NK Kondelule JK Lainjo B Kelly R The Social Dynamics of HIV Transmission as Reflected through Discordant Couples in Rural Uganda Aids 1995 14 745 750 10.1097/00002030-199507000-00012 7546420

[B45] Senkoro KP Boerma JT Klokke AH Ng'weshemi JZL Muro AS Gabone R Borgdorff MW HIV incidence and HIV-associated mortality in a cohort of factory workers and their spouses in Tanzania, 1991 through 1996 Journal of Acquired Immune Deficiency Syndromes 2000 14 194 202 10737435 10.1097/00126334-200002010-00012

[B46] Hugonnet S Mosha F Todd J Mugeye K Klokke A Ndeki L Ross D Grosskurth H Hayes R Incidence of HIV infection in stable sexual partnerships: A retrospective cohort study of 1802 couples in Mwanza Region, Tanzania Journal of Acquired Immune Deficiency Syndromes 2002 14 73 80 10.1097/00126334-200205010-00010 12048366

[B47] Celum C Wald A Lingappa JR Magaret AS Wang RS Mugo N Mujugira A Baeten JM Mullins JI Hughes JP Acyclovir and Transmission of HIV-1 from Persons Infected with HIV-1 and HSV-2 New England Journal of Medicine 2010 14 427 439 10.1056/NEJMoa0904849 20089951 PMC2838503

[B48] Bunnell R Opio A Musinguzi J Kirungi W Ekwaru P Mishra V Hladik W Kafuko J Madraa E Mermin J HIV transmission risk behavior among HIV-infected adults in Uganda: results of a nationally representative survey Aids 2008 14 617 624 10.1097/QAD.0b013e3282f56b53 18317003

[B49] Haaland RE Hawkins PA Salazar-Gonzalez J Johnson A Tichacek A Karita E Manigart O Mulenga J Keele BF Shaw GM Inflammatory Genital Infections Mitigate a Severe Genetic Bottleneck in Heterosexual Transmission of Subtype A and C HIV-1 PLoS Pathogens 2009 14 10.1371/journal.ppat.1000274 PMC2621345 19165325

[B50] Central Statistical Office Zambia HIV Prevalence Rates Decline 2007 http://www.zamstats.gov.zm/media/hiv_prevalence_rates_decline.pdf

[B51] UNAIDS/WHO Epidemiological Fact Sheet on HIV and AIDS, 2008 Update: Rwanda 2008 UNAIDS

[B52] Hawkins K Price N Mussa F Milking the cow: Young women's construction of identity and risk in age-disparate transactional sexual relationships in Maputo, Mozambique Global Public Health 2009 14 169 182 10.1080/17441690701589813 19333807

[B53] Hunter M The Materiality of Everyday Sex: Thinking Beyond 'Prostitution' East African Studies 2002 14 99 120

[B54] Luke N Economic status, informal exchange, and sexual risk in Kisumu, Kenya Economic Development and Cultural Change 2008 14 375 396 10.1086/522896 PMC4297648 25605976

[B55] Nobelius AM Kalina B Pool R Whitworth J Chesters J Power R "You Still Need to Give Her a Token of Appreciation'': The Meaning of the Exchange of Money in the Sexual Relationships of Out-of-School Adolescents in Rural Southwest Uganda Journal of Sex Research 2010 14 490 503 10.1080/00224499.2010.494776 20628949

[B56] Konings E Bantebya G Carael M Bagenda D Mertens T Validating population surveys for the measurement of HIV/STD prevention indicators Aids 1995 14 375 382 7794542

[B57] Plummer ML Ross DA Wight D Changalucha J Mshana G Wamoyi J Todd J Anemona A Mosha FF Obasi AIN Hayes RJ "A bit more truthful": the validity of adolescent sexual behaviour data collected in rural northern Tanzania using five methods Sexually Transmitted Infections 2004 14 49 56 10.1136/sti.2004.011924 PMC1765853 15572640

[B58] Meyerson B Robbins A Koppenhaver T Fleming D TCM Exposure and HIV Related Knowledge, Attitudes and Practices from the 2003 Makgabaneng Listenership survey in Botswana 2003 Botswana: Policy Resource Group http://www.policyresourcegroup.com/pdfs/Botswana_TCM_Evaluation.pdf

[B59] Obbo C HIV Transmission through social and geographical networks in Uganda Social Sciences and Medicine 1993 14 949 955 10.1016/0277-9536(93)90086-J 8480240

[B60] Parker W Makhubele MB Ntlabati P Connolly C Concurrent sexual partnerships amongst young adults in South Africa: Challenges for HIV prevention communication 2007 Johannesburg, South Africa: Centre for AIDS Development, Research and Evaluation http://www.cadre.org.za/node/140

[B61] Rweyemamu D One Love Connect. Protect. Respect: Multiple And Concurrent Sexual Partnerships Among Youth In Tanzania. A research study commissioned by Femina HIP in preparation for a regional youth MCP campaign 2008

[B62] Standing, Kisekka Sexual behavior and AIDS in Sub-Saharan Africa--An annotated bibliography 1989 ODA (UK)

[B63] Talle A Desiring difference: risk behavior among young Maasai men Young People at Risk: Fighting AIDS in Northern Tanzania 1995 Copenhagen: Scandinavian University Press

[B64] Gregson S Mushati P White PJ Mlilo M Mundandi C Nyamukapa C Informal confidential voting interview methods and temporal changes in reported sexual risk behaviour for HIV transmission in sub-Saharan Africa Sexually Transmitted Infections 2004 14 36 42 10.1136/sti.2004.012088 PMC1765846 15572638

[B65] Langhaug LF Sherr L Cowan FM How to improve the validity of sexual behaviour reporting: systematic review of questionnaire delivery modes in developing countries Tropical Medicine & International Health 2010 14 362 381 20409291 10.1111/j.1365-3156.2009.02464.x PMC3321435

[B66] Poulin M Reporting on first sexual experience: The importance of interviewer-respondent interaction Demographic Research 2010 14 237 287 10.4054/DemRes.2010.22.11 20357897 PMC2847279

[B67] Xu HW Luke N Zulu EM Concurrent sexual partnerships among youth in urban Kenya: Prevalence and partnership effects Population Studies-a Journal of Demography 2010 14 247 261 10.1080/00324728.2010.507872 PMC3366482 20865631

[B68] Eaton J Hallett TB Garnett G Concurrent Sexual Partnerships and Primary HIV Infection: A Critical Interaction AIDS and Behavior 2010 10.1007/s10461-010-9787-8 PMC3520057 20890654

[B69] Goodreau SM Cassels S Kasprzyk D Montañ DE Greek A Morris M Concurrent partnerships, Acute Infection and Epidemic Dynamics in Zimbabwe AIDS and Behavior 2010 10.1007/s10461-010-9858-x PMC3394592 21190074

[B70] Johnson LF Dorrington RE Bradshaw D Pillay-Van Wyk V Rehle TM Sexual behaviour patterns in South Africa and their association with the spread of HIV: Insights from a mathematical model Demographic Research 2009 14 289 339 10.4054/DemRes.2009.21.11

[B71] Morris M Kretzschmar M Concurrent partnerships and transmission dynamics in networks Social Networks 1995 14 299 318 10.1016/0378-8733(95)00268-S

[B72] Morris M Kretzschmar M Concurrent partnerships and the spread of HIV Aids 1997 14 641 648 10.1097/00002030-199705000-00012 9108946

[B73] Morris M Kretzschmar M A Micro-simulation study of the effect of concurrent partnerships on HIV spread in Uganda Math Pop Studies 2000 14 109 133 10.1080/08898480009525478

[B74] Kretzschmar M Morris M Measures of concurrency in networks and the spread of infectious disease Mathematical Biosciences 1996 14 165 195 10.1016/0025-5564(95)00093-3 8718707

[B75] Halperin DT Epstein H Why is HIV prevalence so severe in Southern Africa? Southern African Journal of HIV Medicine 2007 19

[B76] Abu-Raddad L Longini I No HIV stage is dominant in driving the HIV epidemic in sub-Saharan Africa Aids 2008 14 1055 1061 10.1097/QAD.0b013e3282f8af84 18520349

[B77] Hollingsworth TD Anderson RM Fraser C HIV-1 transmission, by stage of infection Journal of Infectious Diseases 2008 14 687 693 10.1086/590501 18662132

[B78] Wawer MJ Gray RH Sewankambo NK Serwadda D Li XB Laeyendecker O Kiwanuka N Kigozi G Kiddugavu M Lutalo T Rates of HIV-1 transmission per coital act, by stage of HIV-1 infection, in Rakai, Uganda Journal of Infectious Diseases 2005 14 1403 1409 10.1086/429411 15809897

[B79] Johnson AM Mercer CH Erens B Copas AJ McManus S Wellings K Fenton KA Korovessis C Macdowall W Nanchahal K Sexual behaviour in Britain: partnerships, practices, and HIV risk behaviours Lancet 2001 14 1835 1842 10.1016/S0140-6736(01)06883-0 11741621

[B80] Laumann E Gagnon J Michael R Michaels S The Social Organization of Sexuality 1994 Chicago: University of Chicago Press

[B81] Reniers G Watkins S Polygyny and the spread of HIV in sub-Saharan Africa: a case of benign concurrency Aids 2009 14 299 307 10.1097/QAD.0b013e328333af03 PMC3122907 19890204

[B82] Boileau C Clark S Assche SBV Poulin M Reniers G Watkins SC Kohler HP Heymann SJ Sexual and marital trajectories and HIV infection among ever-married women in rural Malawi Sexually Transmitted Infections 2009 14 I27 I33 10.1136/sti.2008.033969 19307337 PMC2654116

[B83] Carael M Ali M Cleland J Nuptiality and risk behaviour in Lusaka and Kampala Afr J Reprod Health 2001 14 83 89 10.2307/3583201

[B84] Kiarie JN Farquhar C Richardson BA Kabura MN John FN Nduati RW John-Stewart GC Domestic violence and prevention of mother-to-child transmission of HIV-1 Aids 2006 14 1763 1769 10.1097/01.aids.0000242823.51754.0c 16931941 PMC3384736

[B85] Kinuthia J Kiarie J Farquhar C Richardson B Nduati R Mbori-Ngacha D John-Stewart G Co-factors for HIV Incidence during Pregnancy and the Postpartum Period CROI Abstract 155 2011 Boston 10.2174/157016210793499213 PMC3372399 20946093

[B86] Kwesigabo G Killewo J Godoy C Urassa W Mbena E Mhalu F Biberfeld G Wall S Sandstrom A Decline in the prevalence of HIV-1 infection in young women in the Kagera region of Tanzania Journal of Acquired Immune Deficiency Syndromes and Human Retrovirology 1998 14 262 268 9495227 10.1097/00042560-199803010-00012

[B87] Mitsunaga TM Powell AM Heard NJ Larsen UM Extramarital sex among Nigerian men - Polygyny and other risk factors Journal of Acquired Immune Deficiency Syndromes 2005 14 478 488 10.1097/01.qai.0000152396.60014.69 16010173

[B88] Munjoma M Kurewa E Mapingure M Mashavave G Chirenje M Rusakaniko S Hussain A Stray-Pedersen B The prevalence, incidence and risk factors of herpes simplex virus type 2 infection among pregnant Zimbabwean women followed up nine months after childbirth BMC Women's Health 2010 14 10.1186/1472-6874-10-2 PMC2819995 20064273

[B89] Weinstein KI Ngallaba S Cross AR Mburu FM Tanzania Knowledge, Attitudes and Practices Survey, 1994 1995 Calverton, Maryland: Bureau of Statistics, Planning Commission and Macro International Inc

[B90] Hudson C Concurrent partnerships could cause AIDS epidemics Int Journal of STD and AIDS 1993 14 349 353 10.1177/095646249300400501 8218510

[B91] Watts CH May RM The Influence of Concurrent Partnerships on the Dynamics of HIV/AIDS Math Biosc 1992 14 89 104 10.1016/0025-5564(92)90006-I 1551000

[B92] Abu-Raddad LJ Patnaik P Kublin JG Dual infection with HIV and malaria fuels the spread of both diseases in sub-Saharan Africa Science 2006 14 1603 1606 10.1126/science.1132338 17158329

[B93] Centers for Disease Control CDC Malaria Map Application 2010 http://cdc-malaria.ncsa.uiuc.edu/

[B94] Stothard JR Chitsulo L Kristensen TK Utzinger J Control of schistosomiasis in sub-Saharan Africa: progress made, new opportunities and remaining challenges Parasitology 2009 14 1665 1675 10.1017/S0031182009991272 19814845

[B95] Teller CH Alva S Reducing Child Malnutrition in Sub-Saharan Africa: Surveys Find Mixed Progress 2008 Population Reference Bureau

[B96] World Health Organization The global burden of disease: 2004 update 2008 Geneva, Switzerland http://www.who.int/healthinfo/global_burden_disease/gbddeathdalycountryestimates_persons_age_2004.xls

[B97] Mishra V Bignami S Greener R Vaessen M Hong R Ghys P Boerma T Assche AV Khan S Rutstein S A study of the association of HIV infection with wealth in sub-Saharan Africa 2007 Calverton, Maryland: Macro International Inc

[B98] Gray RH Wawer MJ Reassessing the hypothesis on STI control for HIV prevention Lancet 2008 14 2064 2065 10.1016/S0140-6736(08)60896-X 18572064

[B99] Potts M Halperin DT Kirby D Swidler A Marseille E Klausner JD Hearst N Wamai RG Kahn JG Walsh J Public health - Reassessing HIV prevention Science 2008 14 749 750 10.1126/science.1153843 18467575 PMC3501984

[B100] Wilson D Halperin DT "Know your epidemic, know your response": a useful approach, if we get it right Lancet 2008 14 423 426 10.1016/S0140-6736(08)60883-1 18687462

[B101] Gershon RRM Vlahov D Nelson KE The Risk of Transmission of HIV-1 through Nonpercutaneous, Nonsexual Modes - a Review Aids 1990 14 645 650 10.1097/00002030-199007000-00006 2204358

[B102] Schmid GP Buve A Mugyenyi P Garnett GP Hayes RJ Williams BG Calleja JG De Cock KM Whitworth JA Kapiga SH Transmission of HIV-1 infection in sub-Saharan Africa and effect of elimination of unsafe injections Lancet 2004 14 482 488 10.1016/S0140-6736(04)15497-4 14962531

[B103] Brodine SK Starkey MJ Shaffer RA Ito SI Tasker SA Barile AJ Tamminga CL Stephan KT Aronson NE Fraser SL Diverse HIV-1 subtypes and clinical, laboratory and behavioral factors in a recently infected US military cohort Aids 2003 14 2521 2527 10.1097/00002030-200311210-00016 14600525

[B104] Brown TM Robbins KE Sinniah M Saraswathy TS Lee V Hooi LS Vijayamalar B Luo CC Ou CY Rapier J HIV type 1 subtypes in Malaysia include B, C, and E AIDS Research and Human Retroviruses 1996 14 1655 1657 10.1089/aid.1996.12.1655 8947304

[B105] Couto-Fernandez JC Veloso C Rachid M Gracie RSG Chequer-Fernandez SL Oliveira SM Arakaki-Sanchez D Chequer PJN Morgado MG Human immunodeficiency virus type 1 (HIV-1) genotyping in Rio de Janeiro, Brazil: assessing subtype and drug-resistance associated mutations in HIV-1 infected individuals failing highly active antiretroviral therapy Memorias Do Instituto Oswaldo Cruz 2005 14 73 78 15867968 10.1590/s0074-02762005000100014

[B106] Eyzaguirre LM Erasilova IB Nadai Y Saad MD Kovtunenko NG Gomatos PJ Zeman VV Botros BA Sanchez JL Birx DL Genetic characterization of HIM strains circulating in Kazakhstan Journal of Acquired Immune Deficiency Syndromes 2007 14 19 23 10.1097/01.qai.0000286598.00313.a6 17514018

[B107] Feng T Zhao G Chen L Wang X Shi X Human immunodeficiency virus type 1 strains epidemic in Shenzhen 2006 14 637 641 17121221

[B108] Lukashov V Cornelissen M Goudsmit J Papuashvilli M Rytik P Khaitov R Karamov E de Wolf F Simultaneous introduction of distinct HIV-1 subtypes into different risk groups in Russia, Byelorussia and Lithuania Aids 1995 14 435 439 7639968

[B109] Sukhanova A Kazennova E Bobkova M Kravchenko A Selimova L Khanina T Bobkov A Pokrovskiĭ V Variants of human immunodeficiency virus type 1, detected in Russia among those infected by the sexual route Vopr Virusol 2004 14 4 7 15017845

[B110] UNAIDS Global report: UNAIDS report on the global AIDS epidemic 2010 2010 Geneva http://www.unaids.org/globalreport/HIV_prevalence_map.htm

